# Abandonment at the Transition from Hospital to Home: Family Caregivers' Experiences

**DOI:** 10.4314/ejhs.v31i3.9

**Published:** 2021-05

**Authors:** Alireza Nikbakht-Nasrabadi, Leila Mardanian-Dehkordi, Fariba Taleghani

**Affiliations:** 1 Department of Medical Surgical, School of Nursing and Midwifery, Tehran University of Medical Sciences, Tehran, Iran; 2 Nursing and Midwifery Care Research Center, Department of Adult Health Nursing, School of Nursing and Midwifery, Isfahan University of Medical Sciences, Isfahan, Iran

**Keywords:** Diabetes, multiple chronic conditions, family caregivers, transitional care, qualitative study, content analysis

## Abstract

**Background:**

People with concurrent chronic conditions face different situations that lead to frequent transferring between the hospital and home. Despite the use of different strategies for improving transitional care, these transferring is associated with different challenges. This article aims to explore family caregivers' experiences of transitional care in diabetes with concurrent chronic conditions.

**Methods:**

This descriptive explorative study was done at university hospitals in two big cities (Isfahan and Tehran) of Iran. The data collection was conducted from November 2018 to February 2020 using deep, semi-structured, and face-to-face interviews which are focused on family caregivers' experiences of transitional care. The researchers continued the sampling until the data saturation. Finally, 15 family caregivers were selected through purposive sampling. Data collection and data analysis were performed concurrently. Data were analyzed through the conventional content analysis method.

**Results:**

Two main themes were identified: unsafe transition (unplanned discharge, inappropriate communication, lack of patient center care, and unavailable healthcare team) and erosive effort (financial burden, psychological stress, physical exhaustion, and lack of supportive sources).

**Conclusion:**

The findings point to the importance of designing a discharge plan and preparing family caregivers before being discharged by healthcare providers. It appears to be essential for health managers and policymakers to pay attention to safe transitional care planning. The establishment of transitional care centers will help to ensure continuity of care. Future research focusing on the design and implementation of an appropriate transitional care model is recommended.

## Introduction

People with Multiple Chronic Conditions (MCC) have multiple (two or more) chronic conditions at the same time (1). After hypertension, diabetes is the most common chronic disease in people with multiple chronic conditions (2). The proportion of people with diabetes who experience several concurrent chronic diseases is about 90% in the United States and Spain (3), 75% in the United Kingdom (4), and more than 50% in the Persian Gulf countries (5). The proportion of these people is more than 50% in Iran, too (6). Because of the increasing number of people with multiple chronic diseases, the management of these conditions has become a big challenge for global health systems (7). Multiple chronic conditions lead to an increased likelihood of transferring between different healthcare providers that it can increase costs. According to the evidence, two-thirds of the healthcare costs are assigned to these people (8). To reduce the hospital length of stay and hospital costs, and to increase patient satisfaction, home-based care has substituted hospital-level care (9). They are often at higher risk of experiencing inadequate care transitions as a result of being unprepared for discharge (10)

To date, our understanding of transitional care has focused on transfers either within acute care organizations or to rehabilitation settings (10, 11). However in some countries such as Iran, patients directly transfer home, and there is not a transitional care center there. Responsibility of caring for these patients with high levels of dependence and disability transfer to the family caregivers (12), they have an essential role in supporting patients, and they are providing a different level of care and support to patients based on their conditions (13). Family is the first and most important source of support in the care of chronic patients in Iran. Family members are committed to tradition, and there is a strong emotional relationship between them, and they have a commitment to each other. This traditional structure is one of the most important sources of patient support (14), which leads to providing clinical assistance, completing formal care services, and emotional support during hospitalization and after discharge (15). Frequent transferring of these patients between the hospital and the home decrease quality of care (16), so transitional care is designed to ensure the continuity of healthcare. It is a complete plan of care with the availability of expert healthcare providers who have enough information about the patient's goals, preferences, and clinical condition (17–18). Managing the care transition during patient transfer between different centers is essential to maintain patient safety (19). Unfortunately, evidence shows that this program is done unsuccessfully (20), and patients and their families face different challenges in this process (21), such as poor outcomes, patients' dissatisfaction, readmission, and avoidable side effects (22). There are several studies concerning transitional care performed in different countries that have focused on the coordination and the continuity of health care services to patients transferring between different locations or different levels of care in the same place (17–18). Other studies conducted in Iran focused on the challenge of effective discharge plans (23). However these studies do not provide information on the experiences of family caregivers of transitional care in diabetes with concurrent chronic conditions. However, transitional care is highly sensitive to variations in context and maybe experienced differently by different populations (11). Patients and caregivers are the ones constant in transitional care and valuable sources of information about the quality of care transition (24). This study aims to explore the experiences of family caregivers of transitional care in diabetes with concurrent chronic conditions.

**Study design and setting**: This descriptive exploratory qualitative study was conducted with a conventional content analysis approach (25). It is a research technique for making replicable and valid inferences from texts (or other meaningful matter) to the contexts of their use (26). The study was carried out at the university hospitals in two big cities (Isfahan as cultural capacity and Tehran as the main capacity and modern city) of Iran.

**Sample and recruitment**: Eligible participants were family caregivers of patients with multiple chronic conditions, with various ages, gender, and educational level, who had experienced a care transition within 3 months of enrollment. 15 family caregivers were selected through purposive sampling.

**Data collection**: Data were collected from November 2018 to February 2020 using in-depth, semi-structured, and face-to-face interviews. Interviews are focused on the experiences of family caregivers of transitional care and held in the participants' preferred locations (either in their home or other else). The researcher asked the participants to describe how they were ready to be discharged and how their needs were addressed. The length of time of the interview varied between 40 and 60 minutes, and the median length was 45 minutes. Data collection and, data analysis continued until reaching data saturation, the point at which no new information or new themes result from additional data collection and analysis (26). Additionally, 15 interviews were conducted in total.

**Data analysis**: In this study, qualitative content analysis method of Granehiem and Lundman (2004) was used for data analysis (25): All interviews were audio-recorded, and transcribed verbatim, then were read through several times to obtain a sense of the whole. At first, researchers independently extracted all meaning units. Then, they discussed, and after resolving the discrepancies, assigned codes to the condensed meaning units that were more abstract. Finally, initial codes were created and compared based on differences and similarities and sorted into 9 sub-themes and 3 main themes (Table 1).

**Table 1:** Themes and sub-themes obtained from data analysis

Themes	Sub-themes
unsafe	Unplanned discharge
transition	inappropriate communication
	lack of patient center care
	unavailable health care team
erosive effort	financial burden
	psychological stress physical exhaustion
	lack of supportive sources

Confirmability, credibility, dependability and transferability were used to assure various aspects of rigor (27). Confirmability was established by registering and reporting various steps of the study. Credibility is enhanced by using prolonged involvement with the data (16 months). To ensure dependability, a limited literature review was conducted at the beginning of the study to avoid bias during data analysis. To facilitate transferability, the researchers tried to explain the character of the research setting and the samples.

**Ethical considerations**: This study was approved by the Ethics Committee of Tehran University of Medical Sciences (IR.TUMS.VCR.REC.1397.567). All participants were informed of the objectives of the study and gave written consent.

## Results

In this study, 15 family caregivers engaged: consisting of nine females and six males. They had different educational levels (from primary through university), and their age range was 29 – 55 years old (Table 2).

**Table 2:** Participants' characteristics

code	age	sex	Educational levels	Realtionship with patient
1	30	male	University	Patient'sboy
2	40	female	University	Patient's girl
3	37	male	University	Patient's boy
4	55	male	High school	Patient's boy
5	35	female	University	Patient's girl
6	47	male	University	Patient's boy
7	29	female	University	Patient's girl
8	49	female	University	Patient's girl
9	21	female	High school	Patient's girl
10	47	female	University	Patient's girl
11	42	female	University	Patient's girl
12	55	female	university	Patient's wife
13	57	female	primary	Patient's wife
14	48	male	University	Patient's boy
15	57	male	University	Patient's boy

The following two themes were obtained through the findings of the present study: **unsafe transition** (unplanned discharge, inappropriate communication, lack of patient center care and unavailable healthcare team) and **erosive effort** (financial burden, psychological stress, and physical exhaustion, and lack of supportive sources).

**Unsafe transition**: This theme points to unsafe transferring of patients from hospital to home. Unplanned discharge, inappropriate communication between healthcare providers and the patients and their families, the lack of patient-centered care and unavailable healthcare team lead to unsafe transition.

**Unplanned discharge**: Participants talk about unplanned discharge, which is sometimes done routinely before the complete treatment, which leads to different problems due to unprepared caregivers.

“There was no special preparation at discharge. They gave us a summary note and advised us to refer to the physician office a week later” (p3).

“I preferred my mother discharged earlier ... but we weren't preparing for discharge, my mother transferred to home with irregular blood sugar, which was high constantly or temporary” (p9).

Patient education is one of the essential components of transitional care that is neglected viewpoints of participants. They told about their need for education and the inadequacy of training:

“They have no enough time for patient education, because of insufficient staff, and workload, or maybe educating the patient and the family is not a priority for them at all ... They do not explain what they want to do for the patient and why they do this. My mother was discharged from the hospital while we did not know what the cause of her fever was or how we should follow up (p2).

Another said that they did not train during discharge:

“They did not train us at the time of discharge, only the doctor said that you should consume these drugs. He did not explain the previous drugs and what we did...” (p6)

**Inappropriate communication**: Inappropriate communication between healthcare providers and the patients and their families was another concern of participants. Participants told about improper and unsympathetic voice tone of staff:

“The doctor was not very patient. He did not answer very well. Maybe he was busy. We couldn't communicate with him.” (p5) “I was too stressed when blood pressure of my mom increased. I went to the nursing station and informed them that my mother was not ok, but they are unsympathetic. Their tone of voice was so cold that my sense got worse ”(p11).

**Lack of patient center care**: Participants talked about routine based care that was performed without careful consideration of the needs of the patient, and providing care was done without attention to the condition of the patient.

“My father complained of stomach pain. He saw a general physician. The doctor prescribed a stomach pill for him without order an endoscopy to see if he had gastric bleeding or not ”(p1).

“Sometimes, background problems are missed and the main problem is ignored, because they don't assess patients carefully. For example, my father had a diabetic foot, and he suffered pain. At the first hospitalization, physicians said that it is neuropathy, the second time, they said that it is cellulitis. Finally, they found that it was a fracture. They repeat a series of interventions routinely, while the patients are different ”(p10).

**Unavailable health care team:** Family caregivers talked about a long time waiting for an appointment with physicians, unavailable call contact in a critical situation, and lack of follow-up.

“All caring intervention finished with discharge. We don't access the health team and there was no follow-up (p14).

Unavailable healthcare team is more tangible for whom that live in a village.

“There is no local hospital in our village. For any question or problem, we should go to the city... when we were calling the hospital and explaining about our patients' health problems, they said: “Bring him to the hospital”... Sometimes we wait a long time to see a physician.” (p1).

**Erosive effort**: This theme indicates that transitional care looks like an erosive effort. This theme contains physical exhaustion, financial burden, psychological stress, and lack of supportive recourse.

**Physical exhaustion:** Physical exhaustion is associated with long-term care. Family caregivers point to the fatigue and physical burden of care that sometimes provide it alone.

“In my opinion, one of the issues that exist is that caregivers themselves are affected by caring, they become exhausted and cannot follow up on their physical problems. I have heart palpation and I do not have the opportunity to visit the doctor” (p2).

“My father had lost his foot to amputation, he cannot move. So, I should move him. I am alone, and it is too difficult for me to move him, sometimes it is truly impossible” (p7).

**Financial burdens:** The costs of repeated diagnostic procedures, drugs, and medical equipment for home care, which is not supported by insurance companies, lead to financial difficulties.

“When fever of my mother shot up high, her consciousness and her breathing is disturbed. We should have a ventilator at home, which was expensive. The mask of noninvasive ventilation was expensive too” (p2).

“My Dad got hospitalized for a short time. He is under the protection of an insurance company, but the cost of a 3-day hospital length of stay was very high. Also, the cost of dressing his foot wound is high too and doesn't protect by the insurance company” (p7).

**Psychological stress**: Family caregivers experienced psychological stress because of the critical and unstable condition of the patients and transferring the responsibility of patient care to them in this situation.

“I feel that I need help. Well, being in this situation needs tolerance. It is so stressful that whom you love is in a bad condition, and you care for whom. It is associated with a psychological problem. I am under tension” (p12).

“Caring responsibility limits my social relationship. I think I am depressed … I feel stress. It is actually difficult to take care of the patient and know that you could not do anything for whom.” (p5).

**Lack of supportive recourse**: Lack of supportive resources was another issue that is experienced by family caregivers. Participants felt the need to support resources and try to find it.

“There were medical pieces of equipment in the hospital, but we don't access them outside the hospital... Sometimes, we need assistance, and we don't know where we can find it. We need affordable services. ” (p13)

“I don't know if there is any place to help us. It is difficult to find the drug, we don't know where we can get it” (p1).

## Discussion

This qualitative study explored the experiences of family caregivers of transitional care in diabetes with concurrent chronic conditions. The findings indicate that they are abundant during the transition from hospital to home. They experience unsafe transition due to unplanned discharge and insufficient education. Mitchell et al. (2018) conducted a study to describe patient and caregiver experiences during care transitions. Their findings showed that providing actionable information is related to safe and manageable care transitions. This finding points to the informational need of caregivers (22). This event was repeated in other studies, too (28,29). Arnautovska et al. (2020) line with the present study said that about 38% of patients do not be prepared for discharge (28). Recently patients face more problems than in the past because of unmet needs before discharge due to shorter hospital stays and earlier hospital discharge (29).

Improper communication between caregivers and health care providers was another finding of this study. Some studies across this study point to the improper communication between the health care team and patients (28–29). But findings of Bucknall et al. (2016) showed that patients experienced augmenting communication between themselves, their families, and health professionals across transitions of care (31). This dissimilarity can be related to the difference in the type, setting, and samples of these studies. But all studies point to the importance of communication between the health care team and patients and their families. However, Scott (2015) said that communication must be standard and perceptible. If it is improved, it would be lead to a reduction in health care errors and readmission (29). The lack of Patient-centered care was one of the concepts in the present study. Fuji et al. (2013) also point to the needs of patients with multiple problems that are not meet. Patientcentered care leads to achieving a positive outcome in these people (32). Also, in this approach, care is provided to patients based on their needs and leads to improved patients outcomes (33). Despite the positive effects of patient-centered care, lack of comprehensive policies, insufficient education, uncontained patient care, missed patients' needs, inappropriate communication, and staff shortages limit patients care (33–34). Unavailable health care team after hospital discharge was another finding of this study. In the study of Bélanger et al. (2018), same as this study, family members stressed that to facilitate their participation in the patient care process, they need to have access to staff who are aware of patients' needs (35). Zammit et al (2017) point to the available health care team as a main factor in successful care management, too (36).

The erosive effort is another finding of the present study. Findings showed that family caregivers face different challenges in the longterm care of patients with MCC, and they experienced financial, physical, and psychological burdens. Tabootwong et al. (2020) said that family caregivers participate in the planning and delivery of care of older patients, and they experience physical, psychological, social, and financial consequences of care. Family caregivers experienced low sleep quality, strain, reduced social interaction (37). Other studies confirmed this finding too (38–39). Health care support is a key point of transition care, but in this study but it is ignored in the present study, and family caregivers did not access to the supportive resource (40). The authors don't find confirming findings in other studies. The experiences of people may be different in different contexts.

This study shows that there is still a gap in the transition from hospital to home. So for a safe transition, it is necessary to provide appropriate conditions for family caregivers participating in transitional care. Health policymakers can facilitate this approach by designing a transitional plan. Care managers can improve the communication skill of health care members through continuous education. Patients' assessment and more supervision in nursing care can guarantee Patient center care. Family caregivers face different challenges by establishing supportive centers, and rotate the caring responsibility can decrease burdens. We suggest that future research should focus on designing a context-based transitional care model.
